# Administration of antioxidants at parturition in Italian Mediterranean lactating buffaloes: findings in the postpartum

**DOI:** 10.1007/s11250-026-05137-z

**Published:** 2026-06-12

**Authors:** Lorenza Frattina, Alice Carbonari, Matteo Burgio, Giovanni Bramante, Olga Maria Andriulo, Edmondo Ceci, Ciro Galdieri, Antonietta D’Onghia, Vincenzo Cicirelli, Annalisa Rizzo

**Affiliations:** 1 Department of Veterinary Medicine, University of Bari Aldo Moro, S.P. per Casamassima km. 3, 70010 Valenzano, BA Italy; 2 Associazione Regionale Allevatori Puglia, Strada Comunale San Nicola 2, 70017 Putignano, BA Italy

**Keywords:** Buffaloes, α-tocopherol, β-carotene, Reproductive performance, Postpartum

## Abstract

The transition period is an extremely critical phase for the Mediterranean lactating buffaloes, characterized by a state of oxidative stress. Oral administration of antioxidants is common. However, the aim of this study was to evaluate whether the intramuscular administration of a vitamin combination, containing α-tocopherol and β-carotene, could improve both milk quality and reproductive performance. The animals were divided into two groups: a group treated, at 24 h postpartum, with a vitamin combination (group D) and a control group (C). For milk quality, concentrations of retinol, α-tocopherol, fat, protein, lactose, Somatic Cell Count (SCC), non-fat dry matter (NFDM), casein, urea, pH, satured fatty acids (SFA), unsaturated fatty acids (UFA), monounsaturated fatty acids (MUFA) and poliunsaturated fatty acids (PUFA) in milk were assessed at 7 and 30 days post-treatment. Reproductive performance was evaluated using two parameters: pregnancy rate and the calving-conception interval. Retinol concentrations did not differ significantly between groups or across time points. α-tocopherol levels were significantly higher in group D compared to group C at T30 (0.35 ± 0.016 vs. 0.27 ± 0.015 mg/100 g; *p* < 0.05), whereas in group C exhibited a significant decrease over time. Fat, SFA, UFA, MUFA, and PUFA levels were significantly elevated in group D relative to group C (*p* < 0.05), while SCC showed a significant reduction in the treated group than the control one (104.4 ± 20.12 vs. 136.6 ± 32.24 × 10³ cells/mL; *p* < 0.05). No significant differences were observed in pregnancy rates; however, the calving-conception interval was significantly shortened in group D (62.23 ± 6.17 vs. 72.88 ± 5.49 days; *p* < 0.01). The obtained results indicate that the parenteral administration of the vitamin combination was associated with changes in selected milk quality parameters and with a shorter calving-conception interval.

## Introduction

The domestic buffalo (*Bubalus bubalis*) is a significant species in rural activities across many countries, especially in the Mediterranean, where it is valued for milk and meat production. Although these animals are resilient and adapt to challenging environmental situations, such conditions can impact their productivity and reproduction, leading to delays in sexual maturity and low conception rates (Perera 2011).

Regarding reproduction, the most critical period and more influenced by changes, which the animals are subjected to, is the transition period that goes from three weeks before to three weeks after calving. During this period, buffaloes undergo profound metabolic, immune, and endocrine changes (Fiore et al. 2017). They must adapt to the physical and biological transition from pregnancy to lactation, alongside environmental changes, such as new rations (with modified ingredients and energy density) and adjustments to housing (Fiore et al. 2017). During this phase, buffaloes typically secrete more energy (fats, proteins, and lactose) in milk than they intake through diet. Consequently, animals experience a notable Negative Energy Balance (NEBAL), which is associated with an increased risk of metabolic disorders and health problems as well as reduced reproductive performance (i.e. days open until first service and conception rate). These changes are linked to an increased energy demand to support milk production and recovery of ovarian activity (Fiore et al. 2014).

Moreover, buffaloes experience a decline in immune defences during the peripartum period due to stress and hormonal fluctuations, making them more susceptible to infections and metabolic issues, such as triglyceride accumulation in the liver, affecting overall health (Contreras et al. 2010). However, metabolic disorders are less prevalent in buffaloes than in dairy cows, due to their different metabolic adaptations and lower milk production (Fiore et al. 2018).

Hormonal imbalances, including those related to a drop in progesterone (P4) and an increase in prepartum oestrogens, trigger the calving process (Beg and Totey 1999). In the postpartum period, the animal must restore the activity of the hypothalamic-pituitary axis for the resumption of gonadotropin secretion (follicle-stimulating hormone -FSH and luteinizing hormone -LH) to re-establish the normal ovarian cycle (Gokuldas et al. 2010).

This physiological process, in turn, could be influenced by other molecules, such as high concentrations of prolactin that make the ovaries refractory to the action of FSH and LH, potentially leading to true anestrus with ovarian inactivity (Das and Khan 2010).

In addition, oxidative stress, defined as the imbalance between free radicals and antioxidants, can damage tissues and negatively affect reproductive health. The main free radicals, known as Reactive Oxygen Species (ROS), include superoxide (O2-), hydrogen peroxide (H2O2), and hydroxyl radical (OH-) (Rizzo et al. 2012), which play a dual role: in controlled amounts, they are essential for physiological processes like vascular regulation and reproduction, but in excess, they can cause cellular damage to the reproductive tract and the conceptus (Saugstad 2000).

Oxidative stress, triggered by factors like dystocia, infections, and nutritional imbalances, can cause problems such as ketosis, mastitis, and infertility (Kumar et al. 2007; Nayyar and Jindal 2010; Youssef et al. 2010).

In addition, oxidative stress, by altering the function of polymorphonucleates (PMN), has a significant impact on breast health and the onset of clinical and subclinical mastitis (Jóźwik et al. 2012). This inevitably leads to a deterioration of the quality of milk with an increase in somatic cells and a reduction in fat, casein and calcium content (Jóźwik et al. 2012). This deterioration in quality leads to a depreciation of milk and a reduction in the processing capacity of milk for dairy products (Sawa et al. 2007).

Antioxidants are crucial molecules for maintaining oxidative balance, stabilizing free radicals, and protecting tissues; they can be enzymatic or non-enzymatic, sourced from diet such as vitamins A, C, and E (Nayyar and Jindal 2010).

Several studies have also shown that the lack of antioxidants induces an increase in somatic cells in milk (SCC), a reduction in dairy yield and the development of spontaneous oxidized taste (SOF) which adversely affects the organoleptic properties of milk-based products (Baldi et al. 2000).

However, in all studies already published in buffaloes, antioxidants are administered orally and the effects of parenteral administration on the considered parameters are unknown.

Parenteral administration could reduce the disadvantages associated with oral vitamin administration. The oral one is subject to marked individual variability in vitamin intake, closely related to the amount of dry matter intake. Furthermore, administration through dietary supplement exposes vitamins to a greater risk of photodegradation and, above all, to the modulating action of the gastrointestinal system, which can negatively affect their actual absorption (Singh et al. 2021).

In contrast, parenteral administration could ensure that the amount of vitamins administered corresponds to that actually absorbed by the animals, eliminating the influence of digestive and environmental factors typical of oral administration. This study aims to evaluate the effectiveness of administering a vitamin supplement (Dalmavital^®^ - Fatro, Italy), containing α-tocopherol (Vitamin E) and β-carotene (a precursor of vitamin A), to buffaloes, immediately postpartum, to improve productive and reproductive performance. Specifically, it examines whether this treatment influences milk quality, also in terms of antioxidant quantity, and reproductive indices as pregnancy rate and calving-conception interval. Given the importance of vitamins during this critical period for buffaloes, it can be assumed that the parenteral administration may improve the parameters tested and, therefore, the economic performance of the farm.

## Materials and methods

### Ethics approval

All procedures were conducted in compliance with institutional guidelines on animal welfare, with prior informed consent signed by the owners and the ethics committee of the Department of Veterinary Medicine at the University of Bari “Aldo Moro” under protocol number n.16/24.

### Animals

The study was conducted from May to September 2024 at the “Zootecnica Pagliosa” farm, located in the Falciano del Massico area (Caserta-Italy), with a total of 210 animals, of which 100 are lactating. In this study, 40 buffaloes, at the time of calving, were enrolled, aged between 2 and 9 years (6.56 ± 2.50 years), weighing between 600 and 700 kg (630 ± 25.2 kg), and with a BCS between 3.5 and 4 on a scale from 1 to 5 (3.68 ± 0.22). Parity ranged from the first to the sixth calving, with a mean parity of 3.78 ± 1.37. The animals were housed in a free-stall system, ensuring adequate space allowance, natural ventilation, and continuous access to fresh water. The housing facilities were designed to promote animal welfare, allowing free movement, resting, and social interaction, in accordance with current animal welfare guidelines. Buffaloes were fed a total mixed ration (TMR) distributed twice daily, formulated to meet or exceed the nutritional requirements of lactating buffaloes. The diet consisted of corn silage, mixed polyphyta hay, alfalfa hay, cornmeal, and a protein concentrate providing an appropriate balance of energy, protein, fiber, minerals, and vitamins. Feed was offered ad libitum, and refusals were monitored daily to ensure consistent intake and avoid feed restriction. The composition of the TMR was adjusted according to physiological status and stage of lactation, in line with farm management practices.

All animals were free from infectious and parasitic diseases and, before the experimental procedures, underwent a general clinical examination to confirm their health status and proper completion of calving.

Sample size was determined using the standard formula for comparing two independent means (Noordzij et al. 2010) assuming a significance level (α) of 0.05 and a statistical power (1 − β) of 80%. Estimates of variance (*σ*) and expected mean difference *(µ₁ − µ₂)* were derived from previously published studies conducted in bovine species under comparable experimental conditions (Khan et al. 2019). Based on these parameters, the sample size calculation yielded a requirement of 20 animals per group, which was considered the minimum adequate number to detect statistically significant differences between the experimental treatments evaluated in the present study.

### Experimental design

Only buffaloes with eutocic deliveries and who had expelled the placenta were included. All animals were randomly divided into two groups using an online randomization tool (Randomizer.org):

- **Group D**: 20 buffaloes that received one administration of Dalmavital ^®^ (Betacarotene 15 mg + alpha-tocopherol acetate 20 mg/ml) (Fatro S.p.a.- Italy) at a dose of 5 ml/100kg body weight, intramuscularly (IM), within 24 h after calving.
- **Group C**: 20 buffaloes that received one administration of saline solution (NaCl) at 0.9%, IM, at the same dosage and time.

At enrollment, the two groups were comparable for age, body weight, BCS and parity distribution. Mean age was 6.5 ± 2.3 years in Group D and 6.62 ± 2.6 years in Group C. Average body weight was 630 ± 24 kg and 630 ± 27 kg in Groups D and C, respectively. The mean BCS was 3.65 ± 0.24 in Group D and 3.70 ± 0.20 in Group C. Parity distribution was similar between groups, with a mean parity of 3.8 ± 1.32 in Group D and 3.7 ± 1.42 in Group C, and comparable proportions of primiparous and multiparous animals.

Milk samples from each animal were collected 7 and 30 days after calving in 15 ml Falcon tubes, and the tubes were promptly frozen at -20 °C. Samples were transported using a sealed container with dry ice to maintain the cold chain until laboratory analysis.

**Fig. 1:**
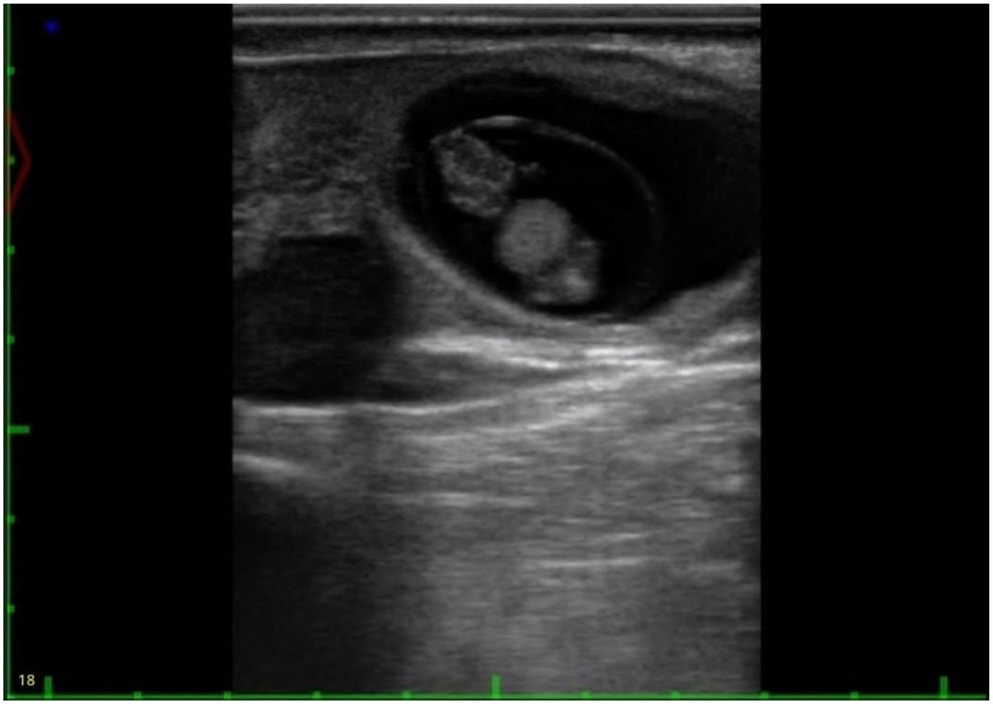
Pregnancy diagnosis in a buffalo through ultrasonographic examination

All the buffaloes included in the study were housed in the maternity ward from two weeks prior to parturition until seven days post-partum. Afterwards, the enrolled animals were separated from the rest of the herd and placed in a group with proven fertile buffalo bulls (1 bull for every 20 buffaloes). The buffaloes were monitored and whenever an operator observed a bull mating, the date of the mating was recorded in a logbook. A complete clinical examination, including rectal examination and ultrasonography, was performed every 30 days, starting from the calving, to confirm pregnancy and diagnose any postpartum pathology. All animals underwent a transrectal ultrasonography (Fig. 1), to estimate the gestational age through fetal measurements, such as crown rump length, abdominal diameter and biparietal diameter of the cranium (Terzano 2012). Data on reproductive indices, including the calving-conception interval and pregnancy rate, were collected within 90 days postpartum.

### Laboratory analysis

The analyses were performed in the laboratory of the Department of Veterinary Medicine in Bari.

Levels of vitamin A, as retinol, and vitamin E, as α-tocopherol, were evaluated in milk samples.

The analysis was performed using HPLC ClinRep^®^ (RECIPE Chemicals + Instruments GmbH) for the determination of vitamin A and vitamin E in serum or plasma. This method was adapted for milk by extracting the fat substance (Rizzo et al. 2013). Two hundred µl of milk sample extract were mixed with 400 µl of internal standard working solution. The resulting precipitate is removed by centrifugation. The supernatant was used for HPLC analysis in an isocratic Agilent 1200 HPLC system, equipped with UV-Vis detector. The samples were separated on a Reversed Phase Cartridge. Instrument setting: Flow rate 0.6 ml/min, Sample loop 20 µl, Column temperature 40 °C. Detector: Wavelength 340 nm after 3 min 295 nm. Retinol and α-tocopherol concentrations were expressed in mg/100 g of extract.

The milk samples collected were also analyzed to assess milk quality parameters, including fat percentage (Fat%), protein percentage (Protein%), lactose percentage (Lactose%), Non-Fat Dry Matter (NFDM), Somatic Cell Count (SCC), urea concentration, pH, and concentration of Saturated Fatty Acids (SFA), Unsaturated Fatty Acids (UFA), Monounsaturated Fatty Acids (MUFA) and Polyunsaturated Fatty Acids (PUFA). The analyses were performed using mid-infrared spectroscopy with a MilkoScan™ 7 and Fossomatic™ 7 for somatic cell counting (Foss, Hilleroed, Denmark).

### Statistical analysis

Data were collected in an Excel spreadsheet and subsequently analyzed using the statistical program SPSS 19 (IBM, NY). Continuous variables were described as mean ± standard error (SE). The Shapiro-Wilk test and the Bartlett test were used to evaluate the normality and homoscedasticity of continuous variables, respectively. Regarding retinol, α-tocopherol and milk quality parameters concentrations, the General Linear Model (GLM) for repeated measures was used, with time (T7, T30) specified as a within-subject factor and treatment (D and C) as a between-subject factor. Each animal was considered as the experimental unit and the treatment × time interaction was included in the model to evaluate differential temporal responses between experimental groups. For the comparison between experimental groups, related to reproductive indices, the Chi-square test was used. For all tests, a p-value < 0.05 was considered statistically significant.

## Results

The drug administered produced no side effects in any animal.

The results of the assays of retinol (vitamin A) and α-tocopherol (vitamin E) concentrations in milk are shown in Table 1.

**Table 1:** Concentrations of retinol (vitamin A) and α-tocopherol (vitamin E), (Mean ± SE), in milk samples from buffaloes treated with Dalmavital at calving (group D) and buffaloes in which saline solution was administered (group C), at 7 days after calving (T7) and 30 days after calving (T30). P-values indicate the effects of time, treatment and time × treatment interaction obtained from a repeated-measures GLM

				*p* values		
		T7	T30	Time	Treatment	Time × Treatment
Retinol (mg/100 g)	Group D	73.00 ± 1.61	70.30 ± 2.33	0.056	0.08	0.058
	Group C	71.50 ± 2.19	68.5 ± 2.18			
α-tocopherol (mg/100 g)	Group D	0.34 ± 0.019	0.35 ± 0.016	0.002	0.032	0.04
	Group C	0.34 ± 0.020	0.27 ± 0.015			

Regarding retinol concentrations, GLM for repeated measures showed no significant main effects of treatment, time or treatment × time interaction, indicating that retinol levels remained comparable between groups and between times (T7 and T30).

For α-tocopherol levels, the analysis revealed a significant effect of treatment, with higher concentrations in group D compared to group C. A significant time effect was also observed, with decreased levels at T30 compared to T7 in group C. The treatment × time interaction was significant, indicating that the temporal pattern of α-tocopherol concentrations differed between groups: while group D maintained stable levels between T7 and T30, group C showed a significant decrease over time.

The findings concerning milk quality parameters are summarized in Table 2.

**Table 2:** Milk quality parameters, (Mean ± SE), in milk samples from buffaloes treated with Dalmavital at calving (group D) and buffaloes in which saline solution was administered (group C), at 7 days after calving (T7) and 30 days after calving (T30): fat percentage (Fat%), protein percentage (Protein%), lactose percentage (Lactose%), non-fat dry matter (NFDM), somatic cell count (SCC), urea concentration, pH, and concentration of unsaturated fatty acids (UFA), monounsaturated fatty acids (MUFA), polyunsaturated fatty acids (PUFA), and saturated fatty acids (SFA). P-values indicate the effects of time, treatment and time × treatment interaction obtained from a repeated-measures GLM

				*p* values		
		T7	T30	Time	Treatment	Time × Treatment
Fat (%)	Group D	8.78 ± 0.94	10.13 ± 1.55	0.76	0.028	0.45
	Group C	6.67 ± 0.55	6.16 ± 1.3			
Protein (%)	Group D	4.72 ± 0.15	4.3 ± 0,10	0.025	0.32	0.69
	Group C	4.75 ± 0.16	4.40 ± 0.21			
Lactose (%)	Group D	3.89 ± 0.16	4.17 ± 0.16	0.007	0.52	0.045
	Group C	4.07 ± 0.06	4.54 ± 0.10			
SCC (×10³ cells/mL)	Group D	62.4 ± 8.25	104.4 ± 20.12	0.031	0.046	0.53
	Group C	84 ± 10.37	136.6 ± 32.24			
NFDM (%)	Group D	10.26 ± 0.21	9.96 ± 0.24	0.87	0.52	0.64
	Group C	10.31 ± 0.25	10.25 ± 0.31			
Casein (%)	Group D	3.72 ± 0.13	3.48 ± 0.12	0.24	0.79	0.91
	Group C	3.74 ± 0.14	3.46 ± 0.19			
Urea (mg/dL)	Group D	41.22 ± 4.35	45.2 ± 2.17	0.22	0.83	0.73
	Group C	41.42 ± 2.10	47.26 ± 3.22			
pH	Group D	6.56 ± 0.11	6.43 ± 0.10	0.3	0.38	0.56
	Group C	6.67 ± 0.06	6.6 ± 0.06			
SFA (g/100 g of milk)	Group D	5.86 ± 0.68	7.33 ± 1.34	0.18	0.03	0.43
	Group C	4.5 ± 0.41	4.48 ± 0.99			
UFA (g/100 g of milk)	Group D	2.76 ± 0.30	2.63 ± 0.21	0.21	0.006	0.042
	Group C	2.04 ± 0.16	1.55 ± 0.29			
MUFA (g/100 g of milk)	Group D	2.61 ± 0.32	2.53 ± 0.25	0.34	0.005	0.036
	Group C	1.89 ± 0.17	1.39 ± 0.32			
PUFA (g/100 g of milk)	Group D	0.18 ± 0.02	0.20 ± 0.02	0.056	0.04	0.007
	Group C	0.15 ± 0.01	0.12 ± 0.02			

Milk quality parameter values showed significant main effect of treatment in fat percentage, with treated animals showing higher fat values compared with controls across both sampling times. The protein percentage showed a significant effect of time, with differences over time within both group D and group C. Lactose percentage exhibited a significant effect of time, driven by an increase from T7 to T30. The treatment × time interaction was also significant, as this temporal increase was evident only in group C, while group D maintained stable lactose levels.

Regarding Somatic cell counts (SCC) the analysis revealed significant effects for both treatment and time, with group D showing lower SCC values than group C, and both groups exhibiting increased counts at T30 compared to T7.

For the values of Non-fat Dry Matter (NFDM), casein, urea, and pH, no significant effects of treatment, time, or treatment × time interaction.

Concerning the milk fatty acid profile, saturated fatty acids (SFA) showed significant treatment effect, with higher concentrations in group D than group C.

While, for unsaturated fatty acids (UFA), monounsaturated fatty acids (MUFA) and polyunsaturated fatty acids (PUFA) significant treatment and treatment × time interaction were observed.

### Reproductive indices

Reproductive indices such as pregnancy rate and calving-conception interval are shown in Table 3. The pregnancy rate did not show statistically significant differences between the treated group and the control group; however, the value observed in the treated group was higher than that of the control group. Conversely, the calving-conception interval demonstrated a statistically significant reduction in group D compared to the group C.

**Table 3:** Reproductive indices expressed as pregnancy rates and calving-conception interval in buffaloes treated with Dalmavital at calving (group D) and buffaloes in which saline solution was administered (group C)

	Pregnancy rate	Calving-conception interval (days)
Group D	13/20 (65%)	62.23 ± 6.17 **
Group C	8/20 (40%)	72.88 ± 5.49 **

In column **: *P* < 0.01

## Discussions

Metabolic stress, which characterizes the transition period in buffaloes, leads to an increase in the production of ROS, not accompanied by a rise in endogenous antioxidants. This condition is known as “oxidative stress” and causes a decline in the productive and reproductive performance of these animals, resulting in a negative economic impact on the farm (Quigley and Drewry 1998).

The present study was conducted to assess whether a vitamin combination, containing α-tocopherol and β-carotene, administered within 24 h after calving could influence milk quality and reproductive indices in buffaloes during the postpartum period.

The vitamin combination was administered within the first 24 h following calving. This timing was chosen because buffaloes experience marked metabolic and physiological changes during this stage. Antioxidant vitamins are essential for supporting reproductive health and reducing the incidence of postpartum diseases (Singh et al. 2021).

The vitamin combination was administered parenterally, specifically via intramuscular injection. This method was chosen because these compounds are photosensitive and quickly lose effectiveness when added to feed. Furthermore, administration via dietary supplement does not allow adequate control of confounding factors related to both the amount of vitamins intake by the animals and the influence of the gastrointestinal tract on vitamin absorption. For this reason, the preferred route of administration is one in which the amount of antioxidant absorption was certain.

To evaluate the efficacy of the treatment, levels of retinol and α-tocopherol were measured in milk samples taken 7 and 30 days after calving, as well as milk quality parameters at the same times. Reproductive indices, in terms of pregnancy rate and the calving-conception interval were also assessed.

The retinol and α-tocopherol levels presented in this study are consistent with the data reported in the literature by Khan et al. (2019). Regarding retinol concentrations, higher levels were observed in the treated group compared to the control group at both 7 and 30 days postpartum, even if no statistically significant. This increase may be attributed to the exogenous administration of β-carotene, which, once converted to retinol in the enterocytes, enters circulation and is directed towards the mammary gland for transfer into the milk (Bouda et al. 1980; Rizzo et al. 2013). Adequate retinol concentrations in milk are important to ensure a nutritionally valuable product. Retinol plays a key role as a coenzyme and antioxidant and is involved in biological processes such as growth, reproduction, vision, and immune function (Garau et al. 2021). This makes milk and dairy products with appropriate retinol content essential for human nutrition (Mattera et al. 2007; Graulet 2014; Garau et al. 2021).

As for α-tocopherol concentrations in milk, a statistically significant increase was observed in the treated group compared to the control group at 30 days postpartum. This increase is likely due to the exogenous administration of α-tocopherol in the vitamin combination. This result is particularly important because it may be associated to an improved mammary health status and may contribute to enhanced milk quality (Smith et al. 1997; Politis 2012). Specifically, α-tocopherol aids polymorphonuclear leukocytes (PMNs) in their bactericidal activity at the mammary level, improving mammary health and reducing somatic cell counts in the milk (Smith et al. 1997). Furthermore, α-tocopherol is capable of lowering the concentration of plasmin in milk, a proteolytic enzyme that, by hydrolyzing α-casein and β-caseins, negatively impacts the coagulating properties of milk and decreases cheese yield (Politis et al. 2004; Politis 2012).

α-Tocopherol also affects the organoleptic properties of milk. Adequate concentrations of this vitamin ensure oxidative stability, maintaining the product’s freshness until consumption and inhibiting auto-oxidation processes that lead to the formation of spontaneous oxidized taste (SOF) (Baldi et al. 2000; Politis 2012).

In addition to its nutritional importance, the high levels of retinol and α-tocopherol observed in the treated group are significant for ensuring proper immunization of calves and reducing the incidence of neonatal diseases. Several studies have demonstrated that elevated concentrations of these vitamins in the milk provided to calves are associated with higher serum IgG levels in the calves (Franklin et al. 1998; Quigley and Drewry 1998).

Moreover, in animals from the control group, there was a statistically significant reduction in the concentrations of α-tocopherol and retinol from day 7 to day 30 postpartum. This decline may be explained by the metabolic demands of lactation, which increase antioxidant consumption needed to protect the body from oxidative damage, resulting in a decrease of these antioxidants in the milk (Sgoifo Rossi et al. 2009).

Regarding the milk quality parameters measured in terms of fat percentage, protein percentage, lactose percentage, NFDM, casein percentage, SCC, urea concentration, pH, and concentration of SFA, UFA, MUFA and PUFA are consistent with those reported in the literature for the buffalo species (Boro et al. 2018; Fiore et al. 2018; Garau et al. 2021; Matera et al. 2023; Pasquini et al. 2018).

Oxidative stress during the immediate postpartum period can negatively affect lipid metabolism in the mammary gland, thereby limiting fatty acid synthesis and secretion. Additionally, an increase in ROS promotes lipid peroxidation, which may lead to a reduction in the levels of unsaturated fatty acids in milk (Jóźwik et al. 2012). The results of this study indicate that the percentage of milk fat and the concentrations of SFA, UFA, MUFA, and PUFA are consistent with previously reported values in the literature for both groups (Garau et al. 2021; Pasquini et al. 2018). However, a statistically significant increase in these parameters was observed in the treated group compared to the control group. Several studies have reported an increase in milk fat percentages in animals that were given dietary α-tocopherol supplementation (Kay et al. 2005; Liu et al. 2008). Similarly, the same increase has also been demonstrated in lactating buffaloes that received dietary vitamin A supplementation (Yadav et al. 2018; Singh et al. 2021).The administration of α-tocopherol and β-carotene likely could enhance antioxidant defenses at the mammary level, preserving membrane integrity and supporting the synthesis and secretion of both saturated and unsaturated fatty acids (Deepak et al. 2024). This suggests that parenteral vitamin supplementation, as already demonstrated for oral addition, was associated with an improvement in milk quality.

Regarding somatic cell count, the results align with values reported in the literature for healthy animals without evidence of mammary inflammation (Costa et al. 2020; Sharif et al. 2007). Previous studies in buffaloes have shown that SCC decreases in animals supplemented with vitamin E and vitamin A (Singh et al. 2021). Vitamin E plays a key role in maintaining neutrophil membrane stability and enhancing phagocytic capacity, while limiting oxidative damage associated with inflammatory responses (Varga et al. 2008; Lee and Han 2018; Deepak et al. 2024). In this study, a statistically significant difference between the groups was observed at 7 and 30 days postpartum, with lower SCC values in the treated group compared to the control group. This reduction may suggest an association between antioxidant supplementation and improvement in milk quality. For the evaluation of reproductive performance, two parameters were considered: pregnancy rate and calving-conception interval. The values of pregnancy rate in both groups were consistent with those reported in the literature for buffaloes (Barile 2005; De Araujo Berber et al. 2002; Neglia et al. 2003). Although no statistically significant difference in pregnancy rate was detected, the treated buffaloes exhibited a higher value compared with the control group. Regarding calving-conception interval, the values observed in both groups were lower than those reported in the literature (Nasr 2017). This could be attributed to the presence of a bull in the herd, which may help stimulate the resumption of ovarian activity. Additionally, natural mating contributes to a reduction in the calving-conception interval and an increase in the pregnancy rate (Moioli et al. 1998; Perera 2008). The reduction in calving-conception interval consequently reduces the calving interval, which leads to a reduction in the declining phase of the lactation curve, resulting in a potential increase of economic returns for farmers.

The results obtained indicate that the administration of β-carotene and α-tocopherol combination led to a statistically significant decrease in calving-conception interval in the treated group compared to the control group. These findings are consistent with previous studies, in which has been reported beneficial effects to enhance granulosa cell survival, improve oocyte quality, increase embryo viability, and reduce oxidative stress-induced pathologies (González-Maldonado et al. 2019; Olson and Seidel 2000; Singh et al. 2021).

To further validate the results of this study, it would be advisable to increase the sample size and evaluate oxidative stress biomarkers of the animals included. In addition, biological covariates such as parity, body condition score and production level were not included as covariates in the statistical models. However, the two experimental groups were comparable at enrollment for the main biological characteristics. Future studies including these variables in the analytical models could help to further clarify their potential contribution to the observed responses on productive and reproductive parameters. Furthermore, it would be interesting to compare the same parameters in animals supplemented via feed with those in animals supplemented via parenteral administration, exposed to the same environmental conditions.

In conclusion, parenteral antioxidant administration immediately postpartum was associated with a shorter calving-conception interval, higher milk retinol and α-tocopherol concentrations, and improvements in selected milk parameters in treated buffaloes, with potential benefits for milk nutritional and technological quality.

## Data Availability

The datasets used in the current study are available from the corresponding author upon reasonable request.
